# Study on Differences of Metabolites among Different *Ganoderma* Species with Comprehensive Metabolomics

**DOI:** 10.3390/jof10080524

**Published:** 2024-07-27

**Authors:** Solongo Khadbaatar, Haiying Bao, Xusheng Gao, Huimin Huo

**Affiliations:** 1 Key Laboratory of Edible Fungi Resources and Utilization, Ministry of Agriculture and Rural Affairs, Jilin Agricultural University, Changchun 130118, China; solongokh@mas.ac.mn (S.K.); gaoxusheng@o.cnu.ac.kr (X.G.); huohuimin1216@163.com (H.H.); 2 Botanical Garden and Research Institute, Mongolian Academy of Science, Ulaanbaatar 13330, Mongolia

**Keywords:** *Ganoderma*, metabolomics, paracetamol, vitamin K3

## Abstract

In traditional Chinese medicine, Ganoderma is a kind of edible and medicinal mushroom, which is widely used because of its significant pharmacological activity. There are many species within the *Ganoderma* genus, each with different material bases and applications. However, detailed studies on these species are still lacking. In this study, we investigated the metabolites of *G. leacontextum* (B), *G. lucidum* (C), *G. tsugae* (S) from Changbai Mountain, and *G. tsugae* (M) from Mongolia using metabolomics. The PCA results indicated minimal differences between M and S, whereas B and S exhibited significant variations. A total of 708 differential metabolites were identified in this study, with steroids, triterpenoids, phenols, and quinones being the major metabolites. Specifically, triterpenoids and steroids were higher in C. Meanwhile, phenolic compounds were more abundant in B. Additionally, quinones were more abundant in M and S. We validated some of the main compounds, and the results showed that paracetamol was most abundant in B, making paracetamol a potential marker for identifying B. Additionally, vitamin K3 was found to be more abundant in M and S, which can serve as a marker for their identification. This study provides new insights and a theoretical basis for the development and utilization of the genus *Ganoderma*.

## 1. Introduction

In traditional Chinese medicine, *Ganoderma* is an important edible and medicinal mushroom that has a history of more than 2000 years in China. Due to its unique pharmacological activity, it has been widely cultivated, and annual output for *Ganoderma* has exceeded 10,000 tons [1,2]. Prior chemical studies have shown that modern pharmacological studies of *Ganoderma* have pharmacological activities such as anti-tumor, antioxidant, immune-enhancing, and tranquilizing effects, as well as the improvement of cardiovascular and cerebral vascular diseases and antiaging effects, among other pharmacological activities [3,4]. These are inextricably linked to a series of active compounds such as polysaccharides, polyphenols, triterpenes, sterols, and quinones [5,6]. However, there are more than 80 species in the genus *Ganoderma* that have been discovered; their metabolic components are different, and their pharmacological activities are also different, but no one has yet conducted a detailed study on these [7].

Primary metabolites of fungi refer to the substances produced by fungi through metabolic activities and are necessary for their growth and reproduction, such as sugars, amino acids, common fatty acids, nucleic acids, and the polymers they form [8,9]. Secondary metabolites of fungi refer to a variety of structurally complex compounds synthesized through complex secondary metabolic pathways before and after the growth of certain fungi to a stable stage, while primary metabolites refer to precursor compounds with a simple structure, clear metabolic pathways, and high yield [10]. There are a wide variety of secondary metabolites that are closely related to human medical products and health care, such as peptides, steroids, alkaloids, and so on. In recent years, the identification and analysis of primary and secondary metabolites using metabolomics have been widely applied. Metabolomics is a newly emerging discipline after genomics, transcriptomics, and proteomics and has been widely used in many fields such as plant and animal metabolism, microbial metabolism, disease diagnosis, drug development, etc. This year, metabolomics technology has been gradually applied to the field of edible and medicinal mushrooms to study their metabolic profiles. Li et al. (2022) used metabolomics to study the metabolic profiles of five different species of *Inonotus hispidus*, and the results showed that there were significant differences among them in polysaccharides, steroids, and phenolic compounds, which provided a theoretical basis for the application of *I. hispidus* [11]. Xue et al. used a metabolomics approach and determined that ganoderic acid distribution was higher at the bottom of the cap’s mediostratum layer and in the stipe shell and context layers at the young bud stage, but this did not reveal other material changes and molecular mechanisms [12].

In this study, the metabolomics of *G. leacontextum*, *G. lucidum*, and *G. tsugae* were investigated to evaluate these three species of *Ganoderma* comprehensively and objectively and to provide a new way of thinking and a theoretical basis for a comprehensive application of the genus.

## 2. Materials and Methods

### 2.1. Materials and Chemicals

**Figure 1:**
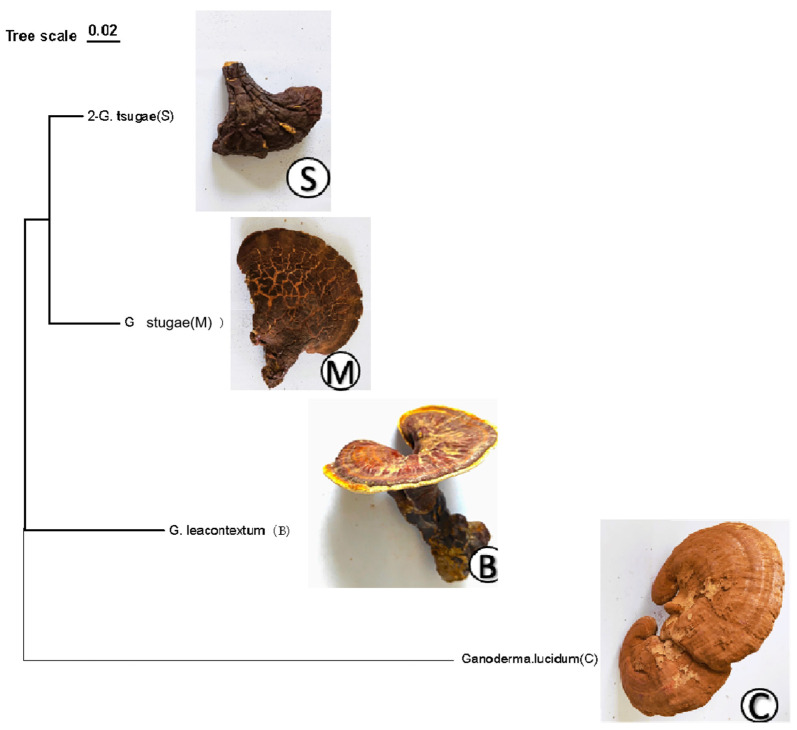
A clustered tree with pictures of four samples of *Ganoderma*.

In this study, four different wild samples of *Ganoderma* were collected. Sample B was *G. leucontextum* taken from the Xizang Autonomous Region, China; sample C was *G. lucidum* taken from Shandong Province, China; sample M was *G. tsugae* taken from Mongolia; and sample S was *G. tsugae* taken from Changbai Mountain, Jilin Province, China. Six randomly selected samples from each sample were preserved at −80 °C until metabolome analysis was carried out (Figure 1). See Supplement S1 for ITS documentation.

To confirm the species identity of the collected samples, molecular genetic analysis was performed. DNA was extracted, and the internal transcribed spacer (ITS) region was amplified using specific primers, ITS1 and ITS4. The amplification products were sequenced, and the sequences were compared with the NCBI database, using BLAST to confirm the species identity. A phylogenetic tree was constructed using the neighbor-joining method with MEGA software (version 11 64-bit), and bootstrap analysis with 1000 replicates was used to assess the confidence of the tree topology.

### 2.2. Metabolite Extraction

Tissues (100 mg) of four *Ganoderma* fruiting body samples were individually ground using liquid nitrogen, and the resultant homogenate was resuspended in prechilled 80% methanol by vortexing. Subsequently, the samples were incubated on ice for 5 min, followed by centrifugation at 15,000× *g* at 4 °C for 20 min. A portion of the supernatant was then diluted to a final concentration of 53% methanol using LC-MS-grade water. The diluted samples were transferred to fresh Eppendorf tubes and subjected to another round of centrifugation at 15,000× *g* at 4 °C for 20 min. Finally, the resulting supernatant was injected into the LC-MS/MS system.

### 2.3. UHPLC-MS/MS Analysis

UHPLC-MS/MS analyses were performed using a Vanquish UHPLC system (Thermo Fisher, Bremen, Germany) coupled with an Orbitrap Q ExactiveTM HF mass spectrometer (Thermo Fisher, Bremen, Germany) at Novogene Co. Ltd. Software (Mass hunter 10.0) (Beijing, China). The samples were injected onto a Hypesil Gold column (100 × 2.1 mm, 1.9 μm) using a 12 min linear gradient at a flow rate of 0.2 mL/min. The eluents for the positive polarity mode were eluent A (0.1% FA in Water) and B (methanol). The eluents for the negative polarity mode were eluent A (5 mM ammonium acetate, pH 9.0) and B (methanol). The solvent gradient was set as follows: 2% B, 1.5 min; 2–85% B, 3 min; 85–100% B, 10 min; 100–2% B, 10.1 min; and 2% B, 12 min. The Q ExactiveTM HF mass spectrometer was operated in positive/negative polarity mode with a spray voltage of 3.5 kV, capillary temperature of 320 °C, sheath gas flow rate of 35 psi, auxiliary gas flow rate of 10 L/min, S-lens RF level of 60, and Aux gas heater temperature of 350 °C.

### 2.4. Data Processing and Metabolite Identification

The raw data files generated by UHPLC-MS/MS were processed using Compound Discoverer 3.1 (CD3.1, Thermo Fisher) to perform peak alignment, peak picking, and quantitation for each metabolite. The main parameters were set as follows: retention time tolerance, 0.2 min; actual mass tolerance, 5 ppm; signal intensity tolerance, 30%; signal/noise ratio, 3; and minimum intensity. Peak intensities were normalized to the total spectral intensity. The normalized data were used to predict the molecular formula based on additive ions, molecular ion peaks, and fragment ions. The peaks were then matched using the mzCloud (https://www.mzcloud.org/ accessed on 1 June 2024), mzVault, and MassList databases to obtain accurate qualitative and quantitative results. Statistical analyses were performed using the statistical software R (R version R3.4.3), Python (Python 2.7.6 version), and CentOS (CentOS release 6.6). When data were not normally distributed, normal transformations were attempted using the area normalization method.

### 2.5. Metabolome Validation

Acetaminophen and menaquinone standards were prepared as single master standards. The standard solution was prepared by taking an appropriate volume of the master standard, and then the working standard solution was sequentially diluted to appropriate concentration with methanol. The standard solutions were detected by UPLC-MS. The concentration of the standard solution was taken as the horizontal coordinate and the peak area as the vertical coordinate to determine the linear range and standard curve. The samples were quantified by calculating linear regression equations (see Supplementary Material S2 for standard curves).

### 2.6. Data Analysis

These metabolites were annotated using the KEGG database (https://www.genome.jp/kegg/pathway.html, accessed on 1 June 2024), the HMDB database (https://hmdb.ca/metabolites, accessed on 1 June 2024), and the LIPID Maps database (http://www.lipidmaps.org/, accessed on 1 June 2024). Principal component analysis (PCA) and partial least squares discriminant analysis (PLS-DA) were performed atSIMCA (SIMCA 14.1) (a flexible and comprehensive software for processing metabolomics data). We applied univariate analysis (*t*-test) to calculate the statistical significance (*p* value). Metabolites with VIP > 1 and *p*-value < 0.05, and fold change ≥ 2 or LogFC ≤ 0.5, were considered differential metabolites. Volcano plots were used to filter metabolites of interest based on log2 (FoldChange) and −log10 (*p*-value) of metabolites using ggplot2 in R language. For clustering heat maps, the data were normalized using z-scores of the intensity areas of differential metabolites and plotted using the Pheatmap package in R language. The correlation between differential metabolites was analyzed using the cor in R language (method = Pearson). Statistically significant correlations between differential metabolites were calculated using the cor. test in R language. Statistical significance was set at *p* < 0.05, and correlation plots were plotted using the corrupt package in R. The functions of these metabolites and metabolic pathways were analyzed using the KEGG database. Metabolic pathway enrichment of differential metabolites was performed, and when the ratio was satisfied by x/n > y/N, the metabolic pathway was considered enrichment. When the *p*-value of the metabolic pathway was <0.05, the metabolic pathway was considered a statistically significant enrichment.

## 3. Results

### 3.1. Phylogenetic Analysis

Phylogenetic analysis based on ITS sequences confirmed the species identity of the four samples. Figure 1 shows the phylogenetic tree constructed from ITS sequences, demonstrating the genetic relationships among the samples collected from different regions. The phylogenetic tree indicates significant genetic differences between sample B (*G. leucocontextum*) and sample C (*G. lucidum*), whereas samples M (*G. tsugae* from Mongolia) and S (*G. tsugae* from Changbai Mountain) are genetically closer.

### 3.2. Method Validation

**Figure 2:**
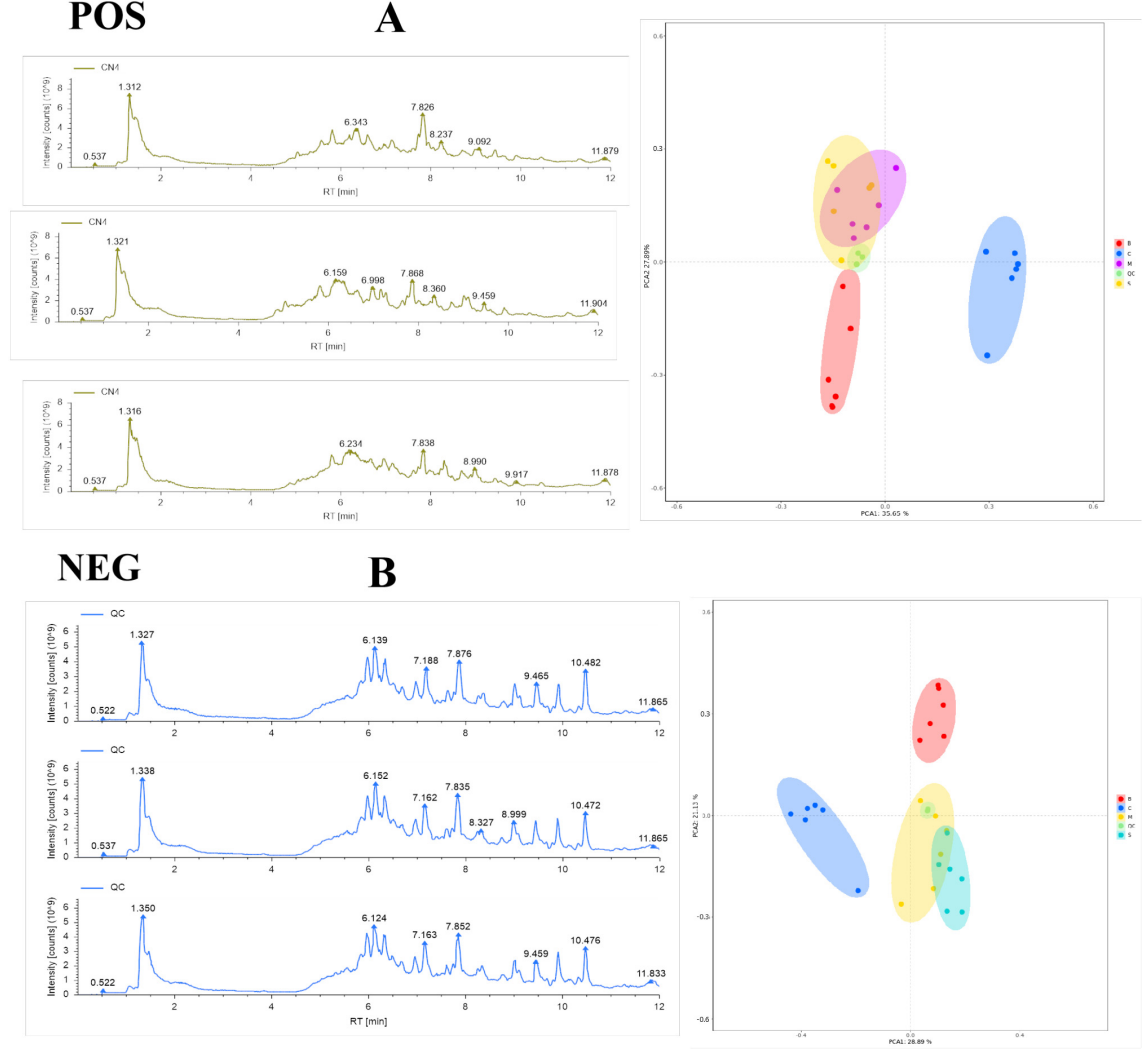
Statistical analysis of PCA for mass spectrometry base peak plots comparing QC samples and overall samples. (**A**) Base peak spectra in positive and negative ion modes; (**B**) PCA scoring plot in positive and negative ion modes.

In order to further investigate the differences in metabolites of different species in *Ganoderma*, the metabolic profiles of four *Ganoderma* samples were analyzed by UHPLC—MS/MS. First, the stability of the UHPLC—MS/MS system was evaluated by comparing the mass spectrometry base peak plots of the quality control (QC) samples with the statistical analysis of principal component analysis (PCA) of the overall samples. The base peaks of the QC samples in positive and negative ion detection modes were compared, and the results showed that the response intensities and retention times of the peaks were basically the same, indicating that the variations caused by the instrumental errors were small and the data quality was reliable. In order to further investigate the differences in metabolites of different *Ganoderma* species, the metabolic profiles of four *Ganoderma* samples were analyzed by UHPLC–MS/MS. First, the stability of the UHPLC–MS/MS system was evaluated by comparing the mass spectrometry base peak plots of the quality control (QC) samples with the statistical analysis of principal component analysis (PCA) of the overall samples (Figure 2A). The base peaks of the QC samples in positive and negative ion detection modes were compared, and the results showed that the response intensities and retention times of the peaks were basically the same, indicating that the variations caused by the instrumental errors were small and the data quality was reliable (Figure 2B).

### 3.3. PCA and OPLS-DA Analysis

**Figure 3:**
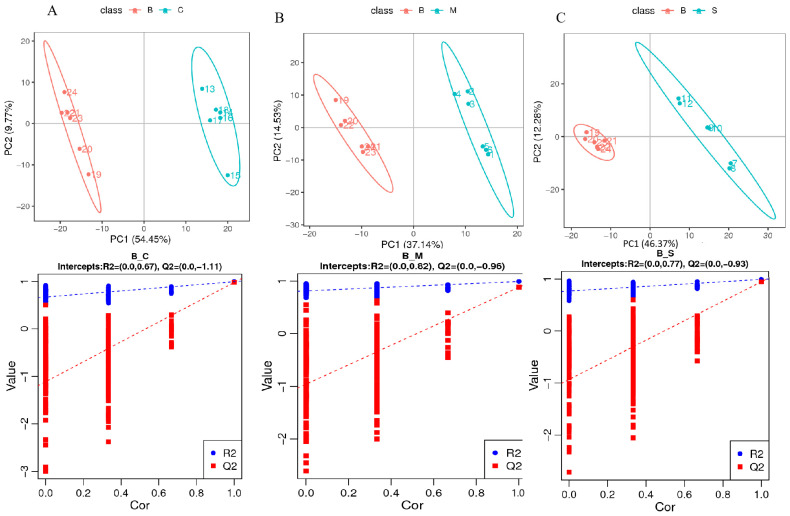
Scatterplots and alignment tests for OPLS_DA models. (**A**) OPLS_DA analysis of B vs. C, (**B**) B vs. M, and (**C**) B vs. S.

In order to confirm the metabolic differences among samples of different species of *Ganoderma*, orthogonal partial least squares discriminant analysis (OPLS-DA) was used to optimize the separation of the population. The two-by-two comparisons of B, C, M, and S samples showed significant metabolic differences between the different groups of samples in the two-by-two comparisons of the first fraction. The OPLS-DA model was constructed with high values of R^2^Y and Q2, as shown in Figure 3, with a good fit and satisfactory predictive ability. In addition, the OPLS-DA model was assessed for possible overfitting using the permutation test. Two hundred permutation tests were conducted. The R^2^ intercepts for samples B and C were 0.67, B and M were 0.82, B and S were 0.77, and the Q^2^ intercepts were −0.11, −0.96, and −0.93, respectively. The above results confirmed that there was no overfitting of the OPLS-DA model and that the model-generated data were plausible.

### 3.4. Comparative Analysis of Differential Metabolites

**Figure 4:**
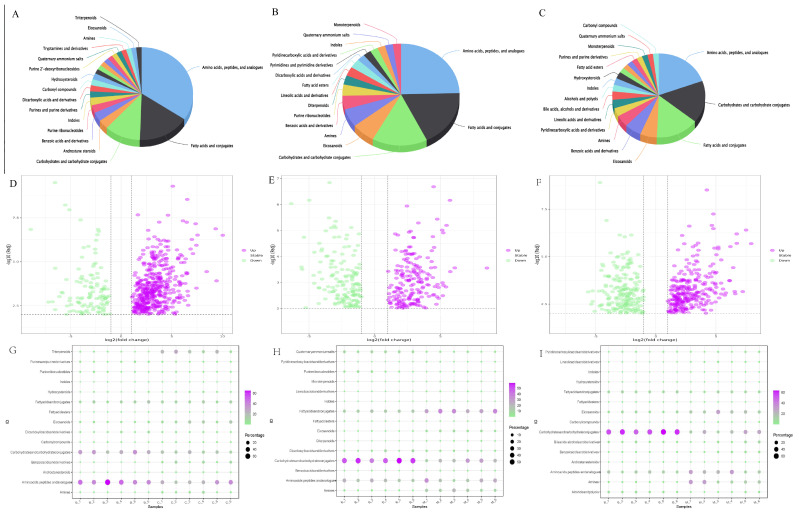
Differential metabolite analysis. (**A–C**) Pie charts showing the relative abundance of metabolite classes in different comparison groups (**A**: B vs. C, **B**: B vs. M, **C**: B vs. S). (**D–F**) Volcano plots displaying significantly different metabolites in different comparison groups (**D**: B vs. C, **E**: B vs. M, **F**: B vs. S). Green dots represent down-regulated metabolites, while purple dots represent up-regulated metabolites. (**G–I**) Bubble charts showing the relative abundance and significance of differential metabolites in different comparison groups (**G**: B vs. C, **H**: B vs. M, **I**: B vs. S). Purple indicates significantly up-regulated, green indicates significantly down-regulated, and bubble size represents relative abundance.

The VIP values obtained from the OPLS-DA model were used to identify different metabolites with biological significance. In this experiment, VIP > 1 was used as a threshold to initially screen for different metabolites among the three groups. One-way analysis of variance (ANOVA) was used to verify whether the metabolites were significantly different. Among the four groups, 708 differential metabolites with VIP values > 1.0 and *p* < 0.05 were identified (Table S1), including 30 fatty acids and conjugates, 21 carbohydrates and carbohydrate conjugates, six androstane steroids, six benzoic acids and derivatives, five purine ribonucleotides, five indoles, four purines and purine derivatives, four dicarboxylic acids and derivatives, four carbonyl compounds, four hydroxysteroids, and so on. In order to better distinguish between them, we used a two-by-two comparison to show the differences. A total of 175 metabolites were downregulated, and 532 metabolites were upregulated in the C sample compared to the B sample. A total of 256 metabolites were downregulated, and 269 metabolites were upregulated in the M sample compared to the B sample. A total of 289 metabolites were downregulated, and 353 metabolites were upregulated in the S sample compared to the B sample (Figure 4D–F). A bubble chart is one of the data statistical analysis charts used to show the relationship between three variables; it can be used to visualize the complex relationship of the data at the same time through the size, color, and position of the bubbles, thus making the data more intuitive. The results of this investigation showed significant differences in carbohydrate conjugates, amino acid peptides, analogs, and triterpenoids between B and C; higher levels of carbohydrate conjugates, amino acid peptides, and analogs in B; and triterpenoids in S. The results of this investigation showed significant differences in carbohydrate conjugates, amino acid peptides, analogs, and triterpenoids between B and carbohydrate conjugates, as well as higher levels of amino acid peptides and analogs and higher levels of triterpenoids in C. In addition, apart from carbohydrate conjugates, amino acids, peptides, and analogs, fatty acids and conjugates differed significantly between B and M. However, carbohydrate conjugates were higher in M compared to B, whereas fatty acids and conjugates were higher in M. It is also interesting to note that between B and S, in addition to the differences between the compounds mentioned above, there were significant differences between amines and eicosanoids, and both are higher in S (Figure 4G–I).

### 3.5. Steroids and Steroid Derivatives Analysis of Four Ganoderma Samples

**Figure 5:**
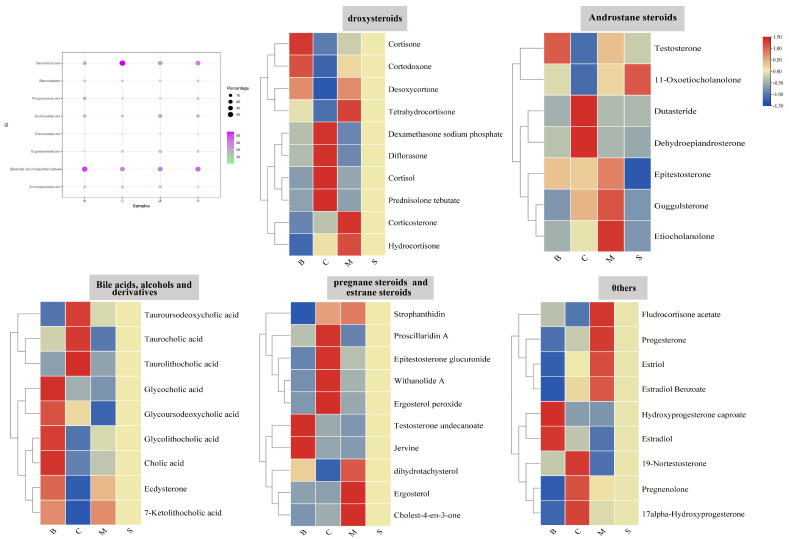
Differential analysis of steroid compounds.

Steroids are recognized as one of the important active ingredients in *G. lucidum*, with important pharmacological activities. A total of 45 steroids and steroid derivatives were detected in this study, which included 10 hydroxysteroids, 9 bile acids, alcohols and derivatives, 7 androstane steroids, 5 pregnane steroids, 4 estrane steroids, 3 steroid lactones, 2 ergostane steroids, and so on. From the abundance bubble plots, it can be seen that there are significant differences among bile acids, alcohols and derivatives, steroid lactones, and hydroxysteroids. For further analysis, each class of steroid was visualized using heat maps, and the results showed that in B cortisone, cortodoxone, glycocholic acid, glycoursodeoxycholic acid, jervine, and estradiol were more abundant in B, whereas cortisol, difluprednate, dutasteride, dehydroepiandrosterone, taurocholic acid, taurolicholic acid, prenylidene saponin A, huperzine lactone A, and pregnenolone were higher in C; in addition to this, the relative abundance of tetrahydrocortisone, corticosterone, hydrocortisone, acetylcholine ketone, ergosterol, cholest-4-en-3-one, fludrocortisone acetate, luteinizing hormone, and estriol was high in M. It is interesting to note that only 11-oxoetiocholanolone had a relatively high abundance in S, while all others were low (Figure 5).

### 3.6. Terpenoid Analysis of Four Ganoderma Samples

**Figure 6:**
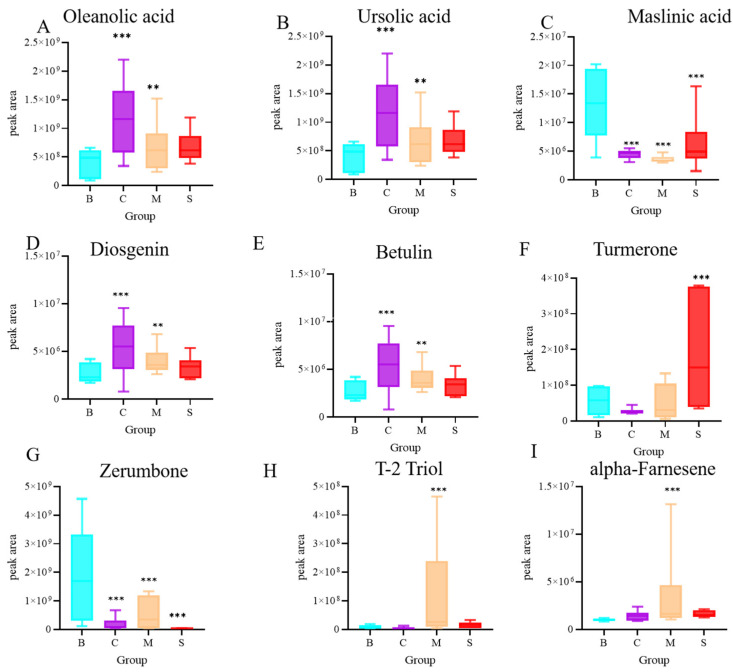
Differential analysis of terpenoids. (**A**) oleanolic acid, (**B**) ursolic acid, (**C**) maslinc acid, (**D**) diosgenin, (**E**) betulin, (**F**) Turncrone, (**G**) Zerumbone, (**H**) T-2 Triol, (**I**) alpha-Fancsene N = 6, ** *p* < 0.02, and *** *p* < 0.001, compared with the B group.

In this study, we performed a classification analysis of terpenoids found in *Ganoderma* species. Terpenoids have a wide range of applications and a high content in C. Terpenoids can be categorized into a number of classes such as monoterpenes, sesquiterpenes, triterpenes, etc., based on their structure and biological activity. These compounds have a wide range of applications and effects in the field of pharmacology. Monoterpenes are commonly used in traditional herbal medicines with antibacterial, anti-inflammatory, analgesic and other pharmacological effects; sesquiterpenes are widely used as anti-tumor, anti-inflammatory, antioxidant, and other drugs, and triterpenes have antiviral, anti-tumor, immune-regulating, and other pharmacological effects, such as ganoderic acid, *G. lucidum* alcohol, and so on. Overall, terpenoids play an important role in the field of pharmacology. Therefore, the analysis of terpenoids in *G. lucidum* is particularly important. The results of this study showed that the relative abundance of oleanolic acid, ursolic acid, and betulin was higher in C, the relative abundance of maslinic acid was higher in S, and the relative abundance of diosgenin was higher in B (Figure 6). In conclusion, triterpenoids overall are higher in B. In addition, sesquiterpenoids also have very important pharmacological activities, and the results of this study show that the relative abundance of T-2 Triol, alpha-Farnesene was higher in M, zerumbone was higher in B, and turmerone was significantly higher in S than in the other three groups.

### 3.7. Phenols Analysis of Four Ganoderma Samples

**Figure 7:**
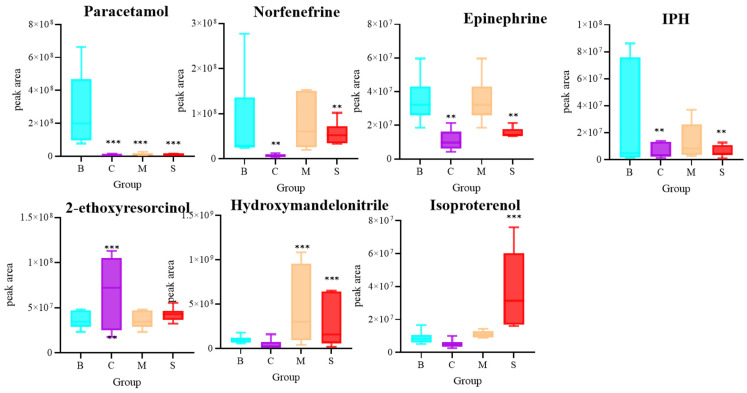
Differential analysis of Phenols. n = 6, ** *p* < 0.02, *** *p* < 0.001, compared with the B group.

Phenolic compounds, as a class of organic molecules with one or more hydroxyl functional groups on the benzene ring, are able to exert very important pharmacological activities in organisms. Their pharmacological activities are mainly due to their intervention in various biochemical processes in organisms, including, but not limited to, antioxidant, anti-inflammatory, anti-microbial, anti-tumor, neuroprotective, and other biological effects. A total of eight phenolic compounds were identified as differential in this study. Paracetamol, norfenefrine, epinephine, and IPH were higher in B, 2-ethoxyesorcinol was higher in C, hydroxymandelonitrile was higher in M, and isoproterenol was higher in S. In summary, the relative abundance of phenolic compounds is higher in B (Figure 7).

### 3.8. Quinone Analysis of Four Ganoderma Samples

**Figure 8:**
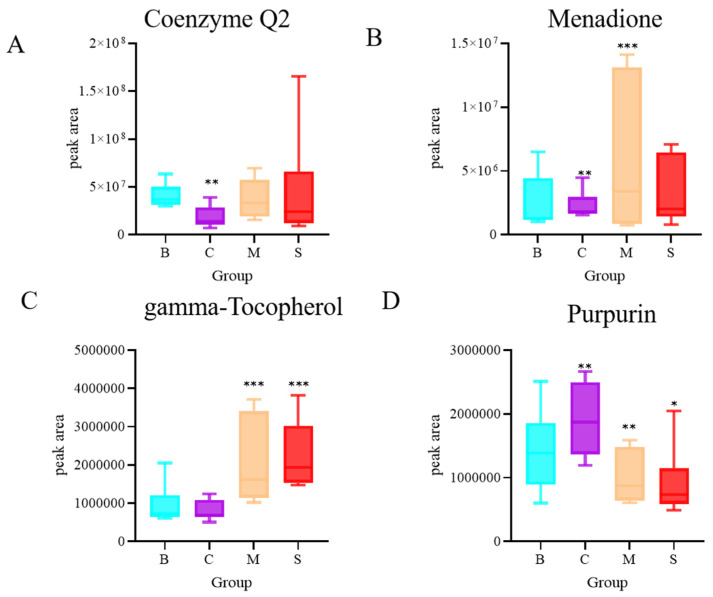
Differential analysis of quinone compounds (**A**) Coenzyme Q2, (**B**) Menadione, (**C**) gamma-Tocopherol, (**D**) Purpurin N = 6, * *p* < 0.05, ** *p* < 0.02, and *** *p* < 0.001, compared with the B group.

Quinone is also an important compound in *Ganoderma* species, and modern pharmacological studies have shown that it has pharmacological activities such as sedation and antioxidant effects, etc. A total of four different quinones were identified in this study, including coenzyme Q2, menadione, gamma-tocopherol, and purpurin, with a relative abundance of gamma-tocopherol and menadione being higher in MS (Figure 8).

### 3.9. KEGG Analysis of Four Ganoderma Samples

**Figure 9:**
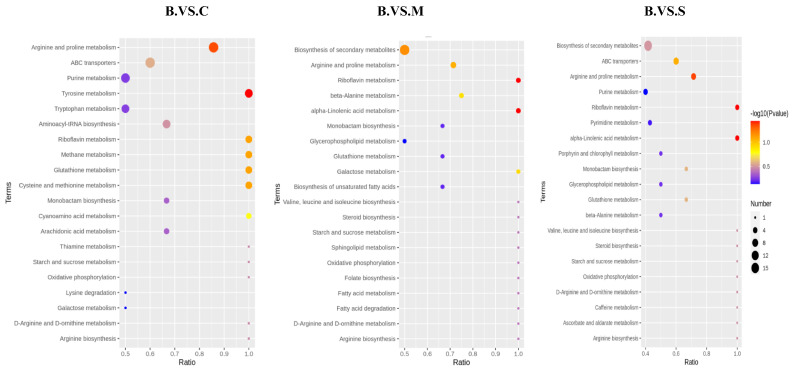
KEGG enrichment bubble diagram of metabolites of different *Ganoderma* species.

In living organisms, different metabolites perform their biological functions in coordination with each other, and pathway-based analyses can help to further understand their biological functions [13,14]. KEGG, known as the Kyoto Encyclopedia of Genes and Genomes, is the main public database of pathways (http://www.genome.jp/kegg/, accessed on 1 June 2024). Pathway analysis allows for the identification of the most important biochemical metabolic pathways and signal transduction pathways in metabolites. The metabolic pathway analysis was used to further understand the differences in the metabolic networks of the four different *Ganoderma* samples, and the differential metabolites were submitted to the KEGG website for metabolic pathway enrichment analysis. The results show that there are large differences in metabolite abundance between them and that this difference is manifested in many metabolic pathways. It is particularly important to distinguish their chemical compositions. Figure 9 shows the enrichment pathways of differential metabolites between them. This includes arginine and proline metabolism, ABC transporters, purine metabolism, biosynthesis of secondary metabolites, riboflavin metabolism, and pyrimidine metabolism and so on.

### 3.10. Metabolome Validation

**Figure 10:**
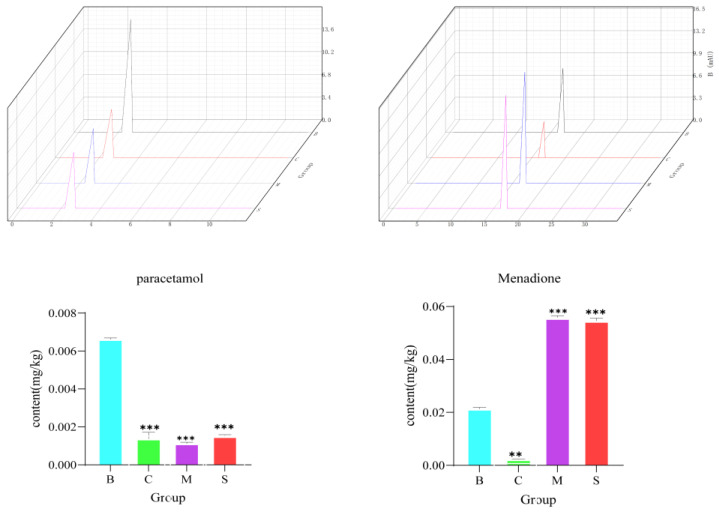
Determination of paracetamol and menadione content (N = 6, ** *p* < 0.02, and *** *p* < 0.001, compared with the B group).

For the above results, our department selected some differential compounds to verify that the results were consistent with the metabolome results (Figure 10). Paracetamol was found to be higher in B and lower—almost absent—in other samples, which was consistent with the metabolomic results, except for vitamin K3, which was found to be higher in M and S, which was also consistent with the metabolomic results.

## 4. Discussion

Traditional Chinese medicine, *Ganoderma*, as an important edible and medicinal mushroom in China, has been documented in the ancient literature for its remarkable pharmacological activities, most of which have been validated [15,16]. The Chinese Pharmacopoeia lists two species of *Ganoderma*, specifically *Ganoderma lucidum*, commonly known as Chi Zhi, and *Ganoderma sinense*, known as Zi Zhi. These species are recognized for their medicinal properties and have been traditionally used in Chinese medicine for their pharmacological benefits, including immune modulation and potential anticancer effects, and were approved as both edible and medicinal species in 2023. However, as there are more than 80 species in the genus *Ganoderma*, the different species of *Ganoderma* lead to different material bases, which result in greater differences in efficacy. Therefore, a comprehensive analysis of the chemical profiles of the species of *Ganoderma* is of great significance to the application of *Ganoderma* species [17,18].

This study conducted a comprehensive analysis of the metabolite composition of four different *Ganoderma* species (*G. leucontextum, G. lucidum, G. tsugae* from Changbai Mountain, and *G. tsugae* from Mongolia) using metabolomics methods. The results showed significant differences in the metabolite composition among these *Ganoderma* species, particularly in steroids, triterpenoids, phenolic compounds, and quinones. These differences not only reveal the chemical characteristics of different *Ganoderma* species but also provide a scientific basis for their potential pharmacological applications.

Firstly, the high content of steroids and triterpenoids in *G. lucidum* is particularly notable. These compounds have various important biological activities in medicine, such as anti-tumor, anti-inflammatory, antioxidant, and immune-modulating effects. Steroids and triterpenoids like ganoderic acid and ganoderol have been extensively studied and have been proven to have significant abilities to inhibit cancer cell growth and enhance immune system function. The high content of these compounds may be related to the genetic characteristics of *G. lucidum* and its growing environment. *G. lucidum* was collected from Shandong, which has a temperate monsoon climate characterized by distinct seasons, warm and humid summers, and cold and dry winters. These climate conditions may promote the accumulation of more secondary metabolites in *Ganoderma* during the growing season, particularly triterpenoids like ganoderic acid and ganoderol [19,20,21].

By contrast, the richness of phenolic compounds in *G. leucontextum* indicates its potential in antioxidant, analgesic, and cardiovascular protective applications. Phenolic compounds have strong antioxidant properties that can neutralize free radicals and reduce oxidative stress damage to cells. *G. leucontextum* was collected from Tibet, which has a plateau climate characterized by low temperatures, strong ultraviolet radiation, and many hours of sunshine [22,23,24]. The high intensity of ultraviolet radiation in high-altitude areas may prompt plants and fungi to synthesize more antioxidant substances to protect themselves from UV damage. Additionally, the low-temperature environment may affect the metabolic pathways of phenolic compounds, causing them to accumulate in the plant.

The high content of quinones in both *G. tsugae* samples indicates their potential in antioxidant and anti-aging applications. Quinones, such as coenzyme Q10 and vitamin K3, have strong antioxidant and anti-aging effects, reducing oxidative stress and inflammation to delay cell aging and protect cell functions. *G. tsugae* from Changbai Mountain grows in a temperate continental monsoon climate, characterized by cold winters and warm, humid summers, with most precipitation occurring in the summer. These climate conditions may favor the accumulation of more antioxidant substances during the growing season. Meanwhile, *G. tsugae* from Mongolia grows in a temperate continental climate, characterized by extremely cold winters and hot, dry summers, with low annual precipitation. These environmental pressures may prompt *G. tsugae* from Mongolia to synthesize more antioxidant substances to cope with oxidative stress caused by extreme temperatures [25,26,27].

From a metabolomics perspective, these significant chemical differences may result from a combination of genetic factors, environmental conditions, and climate characteristics. Each *Ganoderma* species synthesizes various secondary metabolites through different metabolic pathways in their specific growing environments, which play vital roles in responding to environmental pressures, competing for resources, and self-protection. For instance, the synthesis of triterpenoids like ganoderic acid and ganoderol may involve multiple enzymes and metabolic pathways, whose expression and activity are regulated by genes and significantly influenced by environmental conditions. *G. lucidum* may possess a unique genome that enables it to synthesize a large amount of triterpenoids under temperate monsoon climate conditions. By contrast, *G. leucontextum* may have a stronger capacity for synthesizing phenolic compounds, which play an essential protective role under plateau climate conditions.

A particularly notable discovery in this study is the identification of paracetamol (acetaminophen) in *G. leucontextum*. Paracetamol has long been considered a synthetic drug, widely used for its analgesic and antipyretic properties. The detection of paracetamol in *G. leucontextum* suggests that this compound may also occur naturally in some medicinal fungi. This finding opens new avenues for understanding the natural biosynthesis of paracetamol and its potential occurrence in other natural sources. It also raises interesting questions about the evolutionary pathways that might have led to the natural production of paracetamol in certain environments.

Moreover, metabolomics studies also show significant metabolite differences within the same species of *Ganoderma* from different regions. For example, although *G. tsugae* from Changbai Mountain and Mongolia are the same species, they exhibit significant differences in metabolite composition due to different growing environments. Changbai Mountain has a humid climate, while Mongolia has a dry climate with extreme temperatures. These environmental differences directly affect the metabolic activities and secondary metabolite synthesis in *Ganoderma*.

These metabolite differences provide important scientific evidence for the application of *Ganoderma* in drug development and health care and also offer new perspectives for understanding the adaptation mechanisms of plants and fungi. For instance, the high content of triterpenoids in *G. lucidum* makes it a valuable resource for developing anti-tumor drugs, while the phenolic compounds in *G. leucontextum* are suitable for developing antioxidants and neuroprotective agents. Additionally, the quinones in *G. tsugae* can be applied in developing anti-aging skincare products, with broad market prospects.

Climate factors play a crucial role in these differences. The climate characteristics of Changbai Mountain include distinct seasons, warm and humid summers, and cold and dry winters, which may help *Ganoderma* accumulate more triterpenoids during the growing season. Meanwhile, the climate of Mongolia is characterized by dryness and extreme temperatures, with extremely cold winters and hot summers. These environmental pressures may prompt *G. tsugae* from Mongolia to synthesize more antioxidant substances to cope with oxidative stress caused by high and low temperatures. Additionally, the plateau climate of Tibet, where *G. leucontextum* grows, features low levels of oxygen and strong UV conditions, which may induce the synthesis of more antioxidant phenolic compounds to protect cells from environmental stress.

However, this study also has some limitations. Firstly, the sample size is relatively small, and future research should expand the sample size and include more *Ganoderma* species to validate and extend these findings. Secondly, we did not conduct detailed analyses of specific environmental factors affecting metabolite composition, such as soil components, microbial communities, and climate conditions, which may significantly influence metabolite synthesis. Future research should combine environmental science and molecular biology methods to explore the impact of environmental factors on the synthesis of *Ganoderma* metabolites.

In conclusion, this study conducted a comprehensive analysis of the metabolite composition of four different *Ganoderma* species using metabolomics methods, revealing their significant differences and potential pharmacological applications. These findings not only provide scientific evidence for the application of *Ganoderma* in medicine and health products but also point the way for future research. By understanding the metabolite composition of different *Ganoderma* species and their relationship with the environment, we can more effectively develop and utilize these valuable natural resources, contributing to human health.

This study deeply explores the reasons for these differences, including the impact of the climate characteristics of the collection sites on metabolite composition, and discusses the pharmacological activities and profound significance of these differences, including the novel discovery of naturally occurring paracetamol in *G. leucontextum*.

## 5. Conclusions

This study conducted a comprehensive analysis of the metabolite composition of four different *Ganoderma* samples (*G. leucontextum*, *G. lucidum*, *G. tsugae* from Changbai Mountain, and *G. tsugae* from Mongolia) using metabolomics methods, revealing significant differences in their chemical characteristics and potential pharmacological applications. The high content of steroids and triterpenoids in *G. lucidum* indicates its potential in anti-tumor and immune-modulating therapies; the abundance of phenolic compounds in *G. leucontextum* suggests its suitability for antioxidant, analgesic, and cardiovascular-protective applications; and the high levels of quinones in both *G. tsugae* samples highlight their potential use in anti-aging and antioxidant products. Additionally, the discovery of paracetamol, traditionally considered a synthetic drug, in *G. leucontextum* opens new avenues for research into natural biosynthesis. Overall, this study provides valuable insights into the metabolite diversity of *Ganoderma* species.

## Data Availability

The raw data supporting the conclusions of this article will be made available by the authors upon request.

## Supplementary Materials

The following supporting information can be downloaded at: https://www.mdpi.com/article/10.3390/jof10080524/s1, Supplement S1: ITS of four samples, Supplement S2: Liquid phase conditions and standard curve of paracetamol and vitamin K3.

## References

[1] El Sheikha A.F. (2022). Nutritional profile and health benefits of *Ganoderma lucidum* “lingzhi, reishi, or mannentake” as functional foods: Current scenario and future perspectives. Foods.

[2] Zeng P., Guo Z., Zeng X., Hao C., Zhang Y., Zhang M., Liu Y., Li H., Li J., Zhang L. (2018). Chemical, biochemical, preclinical and clinical studies of *Ganoderma lucidum* polysaccharide as an approved drug for treating myopathy and other diseases in China. J. Cell. Mol. Med..

[3] Luz D.A., Pinheiro A.M., Fontes-Júnior E.A., Maia C.S.F. (2023). Neuroprotective, neurogenic, and anticholinergic evidence of *Ganoderma lucidum* cognitive effects: Crucial knowledge is still lacking. Med. Res. Rev..

[4] Wang L., Li J.-Q., Zhang J., Li Z.-M., Liu H.-G., Wang Y.-Z. (2020). Traditional uses, chemical components and pharmacological activities of the genus Ganoderma P. Karst.: A review. RSC Adv..

[5] Seweryn E., Ziała A., Gamian A. (2021). Health-promoting of polysaccharides extracted from *Ganoderma lucidum*. Nutrients.

[6] Fang H., Li X., Lin D., Wang L., Yang T., Yang B. (2023). Inhibition of intrarenal PRR-RAS pathway by *Ganoderma lucidum* polysaccharide peptides in proteinuric nephropathy. Int. J. Biol. Macromol..

[7] Blundell R., Camilleri E., Baral B., Karpiński T.M., Neza E., Atrooz O.M. (2023). The phytochemistry of ganoderma species and their medicinal potentials. Am. J. Chin. Med..

[8] Saleh A.M., Abdel-Mawgoud M., Hassan A.R., Habeeb T.H., Yehia R.S., AbdElgawad H. (2020). Global metabolic changes induced by arbuscular mycorrhizal fungi in oregano plants grown under ambient and elevated levels of atmospheric CO_2_. Plant Physiol. Biochem..

[9] Plett K.L., Wojtalewicz D., Anderson I.C., Plett J.M. (2024). Fungal metabolism and free amino acid content may predict nitrogen transfer to the host plant in the ectomycorrhizal relationship between *Pisolithus* spp. and Eucalyptus grandis. New Phytol..

[10] Xu D., Xue M., Shen Z., Jia X., Hou X., Lai D., Zhou L. (2021). Phytotoxic Secondary Metabolites from Fungi. Toxins.

[11] Li Z., Bao H. (2022). Comparative analysis of metabolic compositions and trace elements of Inonotus hispidus mushroom grown on five different tree species. ACS Omega.

[12] Xue Y., Li X.W., Li Z.Y., Zeng Z.P., Zhang F.S., Li A.P., Qin X.M., Peng B. (2015). UPLC/Q-TOF MS and NMR plant metabolomics approach in studying the effect of growth year on the quality of *Polygala tenuifolia*. Yao Xue Xue Bao = Acta Pharm. Sin..

[13] Dar M.A., Arafah A., Bhat K.A., Khan A., Khan M.S., Ali A., Ahmad S.M., Rashid S.M., Rehman M.U. (2022). Multiomics technologies: Role in disease biomarker discoveries and therapeutics. Brief. Funct. Genom..

[14] Huang X., Su B., Li M., Zhou Y., He X. (2023). Multiomics characterization of fatty acid metabolism for the clinical management of hepatocellular carcinoma. Sci. Rep..

[15] Du Y., Tian L., Wang Y., Li Z., Xu Z. (2024). Chemodiversity, pharmacological activity, and biosynthesis of specialized metabolites from medicinal model fungi *Ganoderma lucidum*. Chin. Med..

[16] Ahmad M.F. (2018). *Ganoderma lucidum*: Persuasive biologically active constituents and their health endorsement. Biomed. Pharmacother..

[17] Peng G., Xiong C., Zeng X., Jin Y., Huang W. (2024). Exploring nutrient profiles, phytochemical composition, and the antiproliferative activity of *Ganoderma lucidum* and *Ganoderma leucocontextum*: A comprehensive comparative study. Foods.

[18] Aiduang W., Kumla J., Srinuanpan S., Thamjaree W., Lumyong S., Suwannarach N. (2022). Mechanical, physical, and chemical properties of mycelium-based composites produced from various lignocellulosic residues and fungal species. J. Fungi.

[19] Fazmiya M.J.A., Sultana A., Rahman K., Heyat M.B.B., Sumbul Akhtar F., Khan S., Appiah S.C.Y. (2022). Current insights on bioactive molecules, antioxidant, anti-inflammatory, and other pharmacological activities of *Cinnamomum camphora* linn. Oxidative Med. Cell. Longev..

[20] Khazdair M.R., Ghorani V., Alavinezhad A., Boskabady M.H. (2018). Pharmacological effects of *Zataria multiflora* boiss l. and its constituents focus on their anti-inflammatory, antioxidant, and immunomodulatory effects. Fundam. Clin. Pharmacol..

[21] Koufakis T., Papanas N., Dimitriadis G., Zebekakis P., Kotsa K. (2020). Interpreting the results of the VERTIS-CV trial: Is this the end of the “class effect” perspective. J. Diabetes.

[22] Peck R.W. (2021). Precision dosing: An industry perspective. Clin. Pharmacol. Ther..

[23] Zenkov N.K., Chechushkov A.V., Kozhin P.M., Kandalintseva N.V., Martinovich G.G., Menshchikova E.B. (2016). Plant phenols and autophagy. Biochemistry.

[24] Xie H., Shen C.Y., Jiang J.G. (2020). The sources of salidroside and its targeting for multiple chronic diseases. J. Funct. Foods.

[25] Souto E.B., Yoshida C.M., Leonardi G.R., Cano A., Sanchez-Lopez E., Zielinska A., Viseras C., Severino P., Silva C.F.D., Barbosa R.D.M. (2021). Lipid-polymeric films: Composition, production and applications in wound healing and skin repair. Pharmaceutics.

[26] Lee H., Vilian A.E., Kim J.Y., Chun M.H., Suh J.S., Seo H.H., Cho S.H., Shin I.S., Kim S.J., Park S.H. (2017). Design and development of caffeic acid conjugated with bombyx mori derived peptide biomaterials for anti-aging skin care applications. RSC Adv..

[27] Dou J., Feng N., Guo F., Chen Z., Liang J., Wang T., Guo X., Xu Z. (2023). Applications of probiotic constituents in cosmetics. Molecules.

