# Supplementary material for: Study on Differences of Metabolites among Different Ganoderma Species with Comprehensive Metabolomics
Source: J Fungi (Basel). 2024 Jul 27;10(8):524. doi: 10.3390/jof10080524 (PMC11355880; doi:10.3390/jof10080524)
Supplement: Supplementary file 1 [file jof-10-00524-s001.zip › Supplement S2.pdf]

1.1 Liquid phase conditions and standard curve of paracetamol

A C18 reverse-phase column (250 mm × 4.6 mm, 5 μm) was used. The mobile phase consisted of water with 0.1% phosphoric acid (A) and methanol (B). The gradient elution program was as follows: 0-5 minutes at 5%B, 5-15 minutes from 5% to 60%B, 15-20 minutes at 60%B, and 20-25 minutes at 5%B. The flow rate was set at 1.0 mL/min, the injection volume was 10 μL, the column temperature was maintained at 25°C, and the detection wavelength was 254 nm. Samples were dissolved in an appropriate solvent, filtered through a 0.45 μm membrane, and then injected. Qualitative and quantitative analyses were performed based on retention time and peak area.

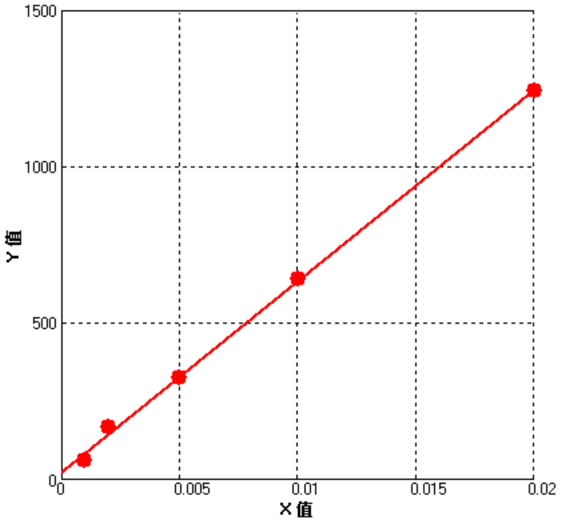

Fig.S1. Standard curve for paracetamol(y = 61088.59185X+24.5927 R^2 = 0.998)

1.2 Liquid phase conditions and standard curve for vitamin K3

Here are the high-performance liquid chromatography (HPLC) conditions for the detection of Vitamin K3 (Menadione): A C18 reverse-phase column (250 mm × 4.6 mm, 5 μm) was utilized. The mobile phase consisted of water with 0.1%

trifluoroacetic acid (TFA) (A) and acetonitrile (B). The gradient elution program was as follows: 0-10 minutes, 30%-90% B; 10-15 minutes, 90% B; and 15-20 minutes, 30% B. The flow rate was set at 1.0 mL/min, the injection volume was 20 μL, the column temperature was maintained at 30°C, and the detection wavelength was 254 nm. Samples were dissolved in an appropriate solvent, filtered through a 0.45 μm membrane, and then injected. Qualitative and quantitative analyses were performed based on retention time and peak area.

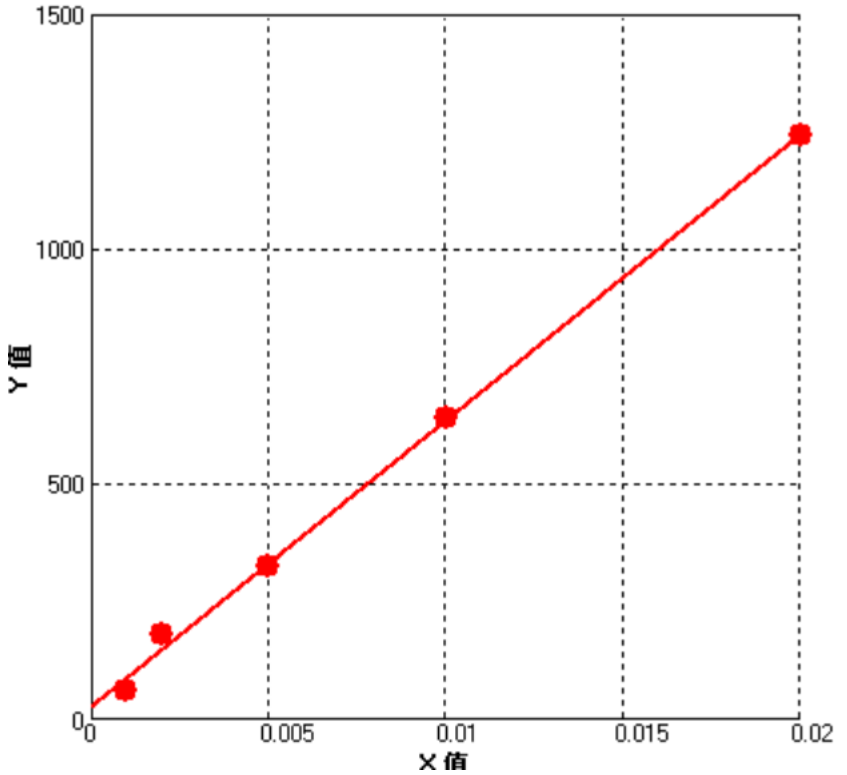

Fig.S2. Standard curve for VK3(y = 610813.21864X+29.05769 $R^2 = 0.998$)
